# Assessing the impacts of imperfect detection on estimates of diversity and community structure through multispecies occupancy modeling

**DOI:** 10.1002/ece3.4023

**Published:** 2018-04-15

**Authors:** David Benoit, Donald A. Jackson, Mark S. Ridgway

**Affiliations:** ^1^ Department of Ecology & Evolutionary Biology University of Toronto Toronto ON Canada; ^2^ Ontario Ministry of Natural Resources and Forestry Peterborough ON Canada

**Keywords:** community ecology, imperfect detection, multispecies occupancy modeling, species richness, stream fish communities

## Abstract

Detecting all species in a given survey is challenging, regardless of sampling effort. This issue, more commonly known as imperfect detection, can have negative impacts on data quality and interpretation, most notably leading to false absences for rare or difficult‐to‐detect species. It is important that this issue be addressed, as estimates of species richness are critical to many areas of ecological research and management. In this study, we set out to determine the impacts of imperfect detection, and decisions about thresholds for inclusion in occupancy, on estimates of species richness and community structure. We collected data from a stream fish assemblage in Algonquin Provincial Park to be used as a representation of ecological communities. We then used multispecies occupancy modeling to estimate species‐specific occurrence probabilities while accounting for imperfect detection, thus creating a more informed dataset. This dataset was then compared to the original to see where differences occurred. In our analyses, we demonstrated that imperfect detection can lead to large changes in estimates of species richness at the site level and summarized differences in the community structure and sampling locations, represented through correspondence analyses.

## 1 INTRODUCTION

Detecting all species present in a given survey is challenging, regardless of sampling effort (Iknayan, Tingley, Furnas, & Beissinger, 2014; Royle, Nichols, & Kery, 2005), yet it is critical for many areas of research in ecology and in management issues (e.g. conservation biology, invasive species). This sampling problem exists whether the target organism is a species of insect (Dorazio, Gotelli, & Ellison, 2011), bird (Ruiz‐Gutierrez, Zipkin, & Dhondt, 2010), mammal (Burton, Sam, Balangtaa, & Brashares, 2012), or fish (Jackson & Harvey, 1997). This issue, more commonly known as imperfect detection, is caused by a number of factors. Variability in abundance is arguably the largest driver of variability in detection, as abundant species are more likely to be encountered within a survey than those that are less common (Royle & Nichols, 2003; Royle et al., 2005). Variability in detection is also heavily influenced by differences among species, as well as differences among individuals of the same species. For example, traits such as size and color may affect the ability of an observer to detect an organism in its natural habitat (Boulinier, Nichols, Sauer, Hines, & Pollock, 1998). Additionally, variation in behavior within a species may make some individuals easier to detect than others. Site‐ and survey‐specific factors have also been shown to influence detectability (Iknayan et al., 2014). For instance, habitat structure at a survey site or inclement weather may impede an observer's ability to detect all individuals present. Rates of detection can also be influenced by variation among observers, with less‐skilled observers being more likely to miss or falsely identify an organism (McClintock, Bailey, Pollock, & Simons, 2010). With such a diverse set of causes, it can easily be understood that imperfect detection is prevalent among ecological studies. Unfortunately, this issue is often left unaddressed.

Ignoring imperfect detection can have a number of negative impacts, most notably on data quality and interpretation. When species are abundant, errors in detection can lead to underestimation of population and range size (Iknayan et al., 2014). This can have serious consequences for those interested in population management, including fisheries and wildlife managers working with economically important species. When species are rare and characterized by low population sizes, imperfect detection leads to false absences and can, ultimately, reduce the accuracy of distribution models and diversity estimates, such as species richness (Dorazio et al., 2011). This is especially concerning when considering the importance of these estimates, as they are not only used in the development of novel ecological theory (e.g., MacArthur & Wilson, 1967) but are also frequently used as information on which conservation and management decisions can be based (Yoccoz, Nichols, & Boulinier, 2001). It is therefore crucial that the accuracy of these estimates be addressed.

A number of statistical methods have been used in the past to estimate species richness while accounting for errors in detection. The most popular of these approaches are the Jackknife and Chao estimators (Burnham & Overton, 1979; Chao, 1984, 1987; Iknayan et al., 2014). Another popular approach is Chao and Jost's (2012) method of rarefaction and extrapolation based on samples of equal coverage, as opposed to size. Despite their frequent use, these approaches have been criticized for confounding occurrence and detection and, ultimately, estimating only the apparent occurrence of undefined species (Kery, 2010). This criticism is due to their failure to address all factors contributing to variation in detectability. Further, these approaches only estimate species richness and do not provide estimations regarding which species may be missing from a location.

An alternative approach that has been put forth to tackle these issues and fully address imperfect detection is that of multispecies occupancy modeling. This form of modeling, developed by Dorazio, Royle, Soderstrom, and Glimskar (2006), can be used to estimate species‐specific occurrence probabilities, while accounting for variability in detection from numerous sources (MacKenzie et al., 2006; Zipkin, DeWan, & Royle, 2009). To use this approach, sites must be sampled repeatedly and the duration of the survey must be kept short enough that species richness can be assumed constant (Dorazio & Royle, 2005). Other major assumptions for this form of modeling are that sample sites remain independent, implying that the detection of a species at one site is independent of detecting that species at other sites and that all species are correctly identified (MacKenzie et al., 2006). One of the major strengths of this form of modeling is its ability to consider rare or difficult‐to‐detect species that may otherwise be ignored due to limited data. This is made possible through hierarchical modeling, whereby parameter estimates for each species are drawn from a common, community‐level distribution, thus, allowing for more precise estimates for rare species (Broms, Hooten, & Fitzpatrick, 2016; Zipkin et al., 2009). Such a community approach can also provide benefits to conservationists and resource managers, as it is much more feasible than conducting numerous single‐species assessments due to reduced costs and time requirements (DeWan & Zipkin, 2010).

Despite their potential, multispecies occupancy models have seen limited use. This is particularly true for taxonomic groups that may have greater inherent problems in sampling, even though some of these groups may be at greater conservation risk. For example, fishes and other freshwater taxa are recognized among the most threatened taxonomic groups (Ricciardi & Rasmussen, 1999) and are also known to have a variety of challenges in their sampling given their habitat and aspects of their biology (Angermeier & Smogor, 1995). Studies examining ecological communities will likely have various degrees of undetected species at one or more locations within the survey data; however, the degree to which such data are missing is rarely considered, let alone formally evaluated. We examine this issue of sampling underestimation through multispecies occupancy modeling. This approach allows us to estimate the number and identity of missing taxa from each sampling location across a range of probability thresholds. Additionally, we propose an approach to evaluate the impact of sampling underestimation on standard analytical methods, such as ordination analysis of ecological communities, given that these multivariate methods are employed widely in ecological studies.

## 2 METHODS

### 2.1 Study area and data collection

We collected presence–absence data of fish communities in Costello Creek, Algonquin Provincial Park, Canada, in July of 2009 and 2015. Sampling consisted of baiting and setting custom triple‐entry minnow traps to be left overnight. The following morning, the traps were retrieved and the captured species were identified. Finescale Dace (*Chrosomus neogaeus*) and Northern Redbelly (*Chrosomus eos*) were grouped into *Chrosomus* spp.*,* as they are similar in appearance, can be difficult to identify in the field and are known to readily hybridize in many locations. All other individuals were classified to the species level. A total of 36 sites were sampled in this manner. Sites were randomly chosen initially along the creek edge and kept constant across both surveys. Sampling was conducted using a triple‐pass system, wherein, each site was sampled three times within each survey period to allow for the calculation of detection probabilities, leading to a total of six replicates at each site. The creek was characterized by two distinct habitat types: a spruce bog with turbid, slow‐moving water and a branch with clear, fast‐flowing water (Figure S1).

In order to ensure that sampling sites remained spatially independent, the home range of the largest fish caught, approximately 150 mm, was mapped on each side of the sampled sites using ArcMAP 10.3.1 (ESRI, 2011). Home range was calculated using an equation described by Minns (1995). Wherever overlap occurred between two sites, the upstream site was removed. This led to the removal of five sites, bringing the total number of sites to 31. Assessing site independence in such a manner also led to the removal of one species from the 2015 dataset, as the Brassy Minnow (*Hybognathus hankinsoni*) was found only at removed sites (Figure S2). It is important to note that although this method of assessing spatial independence may introduce bias into our estimations, sensitivity analyses of site selection were not conducted. Understanding the impacts of site selection was not a goal of this research; however, this methodology does highlight the importance of considering variation in the spatial range of community members.

### 2.2 Multispecies occupancy modeling

The hierarchical community‐model framework used in this study was developed by Dorazio and Royle (2005). Code made available by Zipkin, Royle, Dawson, and Bates (2010) was used as a base for modeling and adapted to this study. To promote model convergence, data from different surveys needed to be pooled. Data from July 2009 and July 2015 were combined to create one dataset. We assumed that sites were at equilibrium, with no immigration or emigration (however, for the purpose of our illustration of how model selection impacts resulting community ordination studies, this assumption is not critical). Any species that were not present in both surveys were removed from the dataset, as their inclusion would have reflected a clear violation of this closure assumption. For this reason, we removed Brassy Minnow, as it was previously removed from one of the datasets when assessing site independence. The final dataset to be used for modeling consisted of 12 species and six sampling replicates at each of the 31 sites. We recognize that the difference between the two time periods may lead to violations of the closure assumption. This issue, and its possible impacts, are further addressed in the Discussion.

Modeling was conducted under the assumption that the occurrence ($\psi_{i,j}$) and detection (*p*_*j*,*k*,*i*_) probabilities varied among species and were influenced by habitat characteristics and survey year, respectively. The occupancy probabilities of each species were modeled dependent on creek width and distance to the sink lake on the logit‐probability scale, as such:

$$\text{logit}\;\left(\psi_{i,j}\right)\;=\;u\left[i\right]\;+\;a1\left[i\right]\cdot \text{width1}\left[j\right]\;+\;a2\left[i\right]\cdot \text{distance1}\left[j\right]$$

In this scenario, *u*[*i*] represents occurrence probabilities for species *i* at point *j*. The coefficient *a1*[*i*] represents the linear effect of creek width on the occurrence of species *i,* and *width1*[*j*] is a vector containing standardized width values for each of the *j* sampled sites. Similarly, the coefficient *a2*[*i*] represents the linear effect of distance to the sink lake on the occurrence of species *i,* and *distance1*[*j*] is a vector containing standardized distance values for each of the *j* sampled sites. Detection was modeled dependent on year of survey:

$$\text{logit}\;\left(p_{j,k,i}\right)\;=\;\nu \cdot \text{YR1}\left[i\right]\cdot \left(1-\text{Year}\left[k\right]\right)\;+\;\nu \cdot \text{YR2}\left[i\right]\cdot \text{Year}\left[k\right]$$

In the above equation, *v.YR1*[*i*] and *v.YR2*[*i*] reflect the detection probability of species *i* in the *k*th survey replicate in the year 2015 and 2009, respectively. This separation is made possible by the indicator function *Year*[*k*]*,* which is used to determine to which year each of the *k* survey replicates matches. Modeling occurrence and detection in such a manner allowed for the calculation of occupancy probabilities for each species at each site. Hierarchical modeling was incorporated into the model by drawing all species parameters from common, community‐level distributions characterized by uninformative priors (Zipkin et al., 2009). We ran three Monte Carlo Markov chains of length 400,000 with a burn‐in of 20,000 and a thinning rate of 200. All analyses were performed using the programs R (R Core Team, 2015) and WinBUGS (Spiegelhalter, Thomas, Best, & Lunn, 2003). Model convergence was assessed using the R‐hat statistic (Gelman & Hill, 2007).

### 2.3 Data analysis

To assess the impacts of imperfect detection, the original dataset was compared to a modified presence–absence matrix that took imperfect detection into account. Occupancy probability values for each species at each site were used to create this “informed dataset.” This was performed by selecting a probability threshold of 95% and comparing it with probability values produced by the model. Any lack of detection for a species at a site with an occupancy probability of 95% or higher was thus considered false absences. These absences, represented by zeroes in the dataset, would then be converted to ones to indicate occurrence. Species richness at the site level was then compared between the two datasets. This was performed by comparing the total number of species observed at each site in the original dataset with the total number of species estimated to be present at each site in the informed dataset. Comparing raw data with model estimates in such a manner allows us to determine the extent to which information is missing from field data. Additionally, this method allows us to highlight the sensitivity of occupancy threshold choice from a management or application perspective (i.e., how threshold choice can influence our understanding of community structure in multivariate analyses and ultimately management practices).

Correspondence analyses were conducted on each dataset to assess community structure (Greenacre, 2007), although this could be performed using any of a variety of ordination methods (see Hirst & Jackson, 2007 for a comparison of methods). Correspondence analysis is a multivariate graphical technique similar to a principal components analysis (PCA). Correspondence analyses are well suited to analyze relationships among categorical variables (Sourial et al., 2010). In this case, we are exploring the relationship between species identity and sampling sites. Interpretation of these ordinations can provide valuable insight on how ecological communities are structured. For example, species that appear close to each other in ordination space are more likely to occupy the same sites, suggesting they may have similar habitat preferences. On the contrary, species that are far from each other in ordinal space are unlikely to occupy the same sites. The same logic can be extended to sampling sites, where sites that appear close together in ordination space are more likely to share species composition, and vice versa. After conducting such an analysis on each dataset, the two ordinations were compared using a resistant‐fit Procrustes analysis. Procrustes analysis is a method that can be used to compare two ordinations by examining the location of landmarks between the two. This method attempts to minimize differences between the two ordinations through translation, rotation, and dilation (Peres‐Neto & Jackson, 2001), effectively providing a multivariate measure of goodness of fit. Deviations between the same landmarks from the two different ordinations, known as residuals, allow us to determine which landmarks differ most between the two ordinations, that is, which species differed most between two ordinations or which sampling sites differed most. Resistant‐fit methods were chosen over the ordinary least‐squares procedures, as they are less likely to show misleading representations of shape differences when one or a few landmarks display large changes in position (Claude, 2008; Marcus, Corti, Loy, Naylor, & Slice, 2013). Procrustes residuals were plotted to determine which sites and species exhibited the most change when imperfect detection was taken into account (Zhao, Shuter, & Jackson, 2008). This process was then repeated for two other arbitrarily chosen probability thresholds (50% and 75%). All analyses were performed using the program R (R Core Team, 2015).

## 3 RESULTS

### 3.1 Multispecies occupancy modeling

A total of 12 species was included in the dataset used for modeling. The included species spanned a number of families, although the majority were in the family Cyprinidae (Table 1). Catch data for each species at each site can be found for both the July 2009 and July 2015 surveys in the Supporting Information (Tables S1 and S2).

**Table 1:** Species detected at least once across all surveys in Costello Creek

Species code	Species	Scientific name	Family
AMNE	Brown Bullhead	*Ameiurus nebulosus*	Ictaluridae
CACO	White Sucker	*Catostomus commersonii*	Catostomidae
CHSP	*Chrosomus* spp.	*Chrosomus*	Cyprinidae
CUIN	Brook Stickleback	*Culaea inconstans*	Gasterosteidae
HYHA	Brassy Minnow^a^	*Hybognathus hankinsoni*	Cyprinidae
LEGI	Pumpkinseed	*Lepomis gibbosus*	Centrarchidae
LUCO	Common Shiner	*Luxilus cornutus*	Cyprinidae
MANA	Northern Pearl Dace	*Margariscus nachtriebi*	Cyprinidae
MIDO	Smallmouth Bass	*Micropterus dolomieu*	Centrarchidae
NOCR	Golden Shiner	*Notemigonus crysoleucas*	Cyprinidae
PEFL	Yellow Perch	*Perca flavescens*	Percidae
SAFO	Brook Trout	*Salvelinus fontinalis*	Salmonidae
SEAT	Creek Chub	*Semotilus atromaculatus*	Cyprinidae

a Species removed from the modeling dataset.

Occupancy probability values produced by the model varied greatly among species and, to a lesser degree, within species, with values ranging from 1.83 × 10^−4^ to 0.999 (Table S3). The mean probability of detection across all sites varied greatly among species, ranging from 0.058 to 0.979 (Table 2). Variation in detection among species was generally much larger than the variation observed between survey years; however, some species did exhibit large changes in detection values between the 2 years. The largest change was seen in the combined Northern Redbelly Dace and Finescale Dace taxon *Chrosomus* spp., where the probability of detection varied by almost 30% between 2009 and 2015.

**Table 2:** Mean detection probability values produced by the model for each species during the July 2009 and July 2015 surveys

Species	Mean probability of detection (2009)	Standard deviation	Mean probability of detection (2015)	Standard deviation	Δ Detection probability
Brook Stickleback	.058	0.057	.206	0.144	.148
Brook Trout	.226	0.154	.173	0.132	−.053
Brown Bullhead	.801	0.042	.619	0.051	−.182
*Chrosomus* spp.	.201	0.054	.481	0.074	.280
Common Shiner	.730	0.048	.776	0.044	.046
Creek Chub	.905	0.030	.979	0.014	.074
Golden Shiner	.744	0.050	.815	0.046	.071
Northern Pearl Dace	.281	0.074	.319	0.079	.038
Pumpkinseed	.494	0.051	.568	0.052	.074
Smallmouth Bass	.175	0.136	.283	0.175	.108
White Sucker	.133	0.040	.100	0.033	−.033
Yellow Perch	.748	0.045	.867	0.035	.119

### 3.2 Species richness

**Figure 1:**
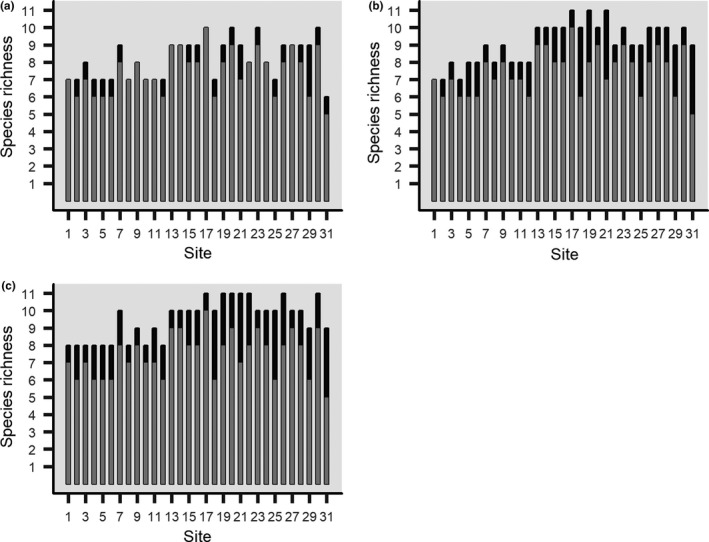
*Species richness at each site when comparing between the standard dataset, represented by gray bars, and estimated species richness at (a) 95%, (b) 75%, and, (c) 50% occupancy probability thresholds, represented by black bars*

Estimates of species richness at the site level underwent changes at all threshold comparisons. When the standard dataset was compared to the 95% threshold, estimated species richness increased by at least one at 20 of the 31 sites (Figure 1a). When the standard dataset was compared to the 75% threshold, an increasing number of sites displayed richness increases of two or more species, with a total of 30 sites experiencing augmentation (Figure 1b). In this scenario, increases in richness were skewed to sites characterized by faster, clearer waters. At the 50% threshold, all sites experienced changes in species richness. Increases in richness varied across sites; however, the largest changes were seen in sites characterized by clear, fast‐flowing waters. The largest change in richness was an addition of four species (Figure 1c).

### 3.3 Community structure

**Figure 2:**
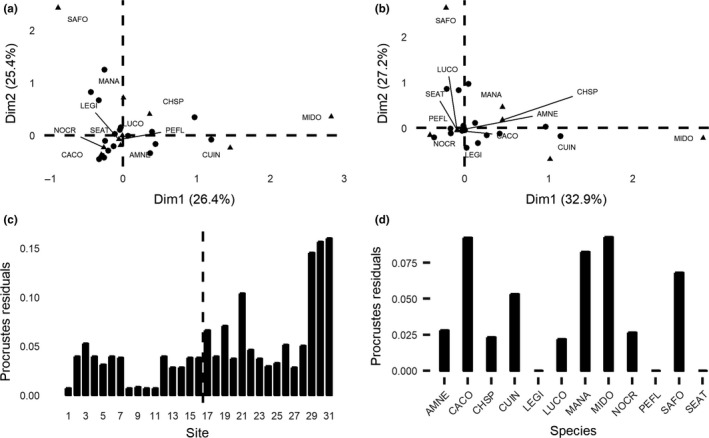
*Results of the correspondence analysis using (a) the standard dataset of fish species presence–absence in Costello Creek and; (b) a dataset informed at the 95% occupancy threshold. Each ordination represents the community structure, with species appearing close together likely occupying the same sites, potentially demonstrating shared habitat preference. Species that are farther apart in the ordination are unlikely to occupy the same sites. For instance, Brook Trout (SAFO) and Smallmouth Bass (MIDO), which are on opposite ends of the ordination, have different habitat preferences and are unlikely to be found together. Differences between the two ordinations, which can be difficult to detect visually, can be displayed as Procrustes residuals. Resistant‐fit Procrustes residuals are displayed for (c) sites, with a dashed line distinguishing the slow‐moving, bog habitat (1*–*16) from the clearer, faster‐flowing waters (17*–31) and (d) each species. *Species codes can be found in Table 1

Community structure, summarized through correspondence analyses, also underwent changes in each threshold scenario. In the standard ordination based on field data, Brook Trout and Smallmouth Bass were located on opposite ends with a large group of species between them. This suggests that Brook Trout and Smallmouth Bass are unlikely to occur at the same sites and may have different habitat preferences, while the other species may be more likely to co‐occur. When comparing this ordination to that of the 95% informed dataset, changes in landmarks are difficult to observe visually. The species and sites responsible for these changes became evident through resistant‐fit Procrustes analyses. When comparing the correspondence analyses of the standard and 95% informed datasets (Figure 2), the largest changes (i.e., the largest residuals in the fit between the two CA ordinations) were seen in Smallmouth Bass and White Sucker. A number of sites also demonstrated large changes (sites 21, 29, 30, and 31). These changes are important to consider, as they have implications for how community relationships will be interpreted, such as which species are most likely to co‐occur and which sites are likely to share species composition. When comparing the standard dataset to the 75% informed dataset (Figure S3), the largest changes among species were seen in Smallmouth Bass and Brook Trout. Further, larger changes tended to occur at sites characterized by clear, fast‐flowing water (sites 17–31). This trend continued when comparing the standard dataset to the 50% informed dataset (Figure S4). As the threshold values decrease (i.e., move from 95% to 50%), changes in the positioning of species in the ordinations become more apparent visually. This coincides with an increase in the residuals and is highlighted by the altered position of Smallmouth Bass and Brook Trout in the 50% informed ordination.

## 4 DISCUSSION

### 4.1 Multispecies occupancy modeling

Although a number of species displayed relatively constant occupancy probability values throughout the entirety of the creek, approximately 50% showed high variation in occupancy between the habitat types. Detection probabilities estimated by the model varied among species (Table 2). These findings, which exemplify the species‐specific nature of detectability, are consistent with previous work performed on reef fish, where it was shown that detection probabilities varied substantially among different species and family groups (MacNeil et al., 2008). Some of this variation is likely attributed to differences in abundance, as commonly observed species in the system tended to have higher probabilities of detection. This trend is to be expected, as both probabilities of occurrence and detection are expected to increase as the abundance of a species increases (Dorazio & Royle, 2005). Variation in detection among species may also be attributed to differences in behavior. For example, fishes have been shown to display differing levels of boldness, a trait that may influence the likelihood of an individual to enter a trap (Bell, 2004). For most species, the mean probability of detection across sites showed little change between survey years; however, this was not the case for *Chrosomus* spp. The large change in detection for this group is due to a large discrepancy in the number of sites where individuals were observed between the two survey years, providing evidence that the closure assumption may have in fact been violated. A number of factors could have led to this result, including population growth or expansion between survey years. Occupancy estimates for *Chrosomus* spp. are therefore likely inaccurate, highlighting the importance of meeting model assumptions. Despite this violation, we believe our results and findings remain relevant, as the purpose of this study was to demonstrate how imperfect detection impacts estimates of diversity and multivariate community representation and not the exploration of specific species relationships.

### 4.2 Species richness

Species richness estimates underwent changes at all threshold levels. At the strictest level, an occupancy probability of 95%, richness estimates changed at about 65% of the sites, with most changes being an addition of one species. However, as threshold values decreased, an increasing number of species were added to an increasing number of sites (Figure 1). At the lowest level, an occupancy probability of 50%, all sites displayed changes in species richness. Although these threshold values were chosen arbitrarily, their consideration is important as they demonstrate the variability imperfect detection can introduce into estimates of species richness. As previously mentioned, species richness estimates are central to both the development of ecological theory and conservation biology and policy (MacArthur & Wilson, 1967; Yoccoz et al., 2001). It is, therefore, crucial that threshold values be given serious thought, as they have the ability to significantly alter richness estimates, and ultimately the development and testing of theory, as well as policy development and implementation.

In Costello Creek, the largest changes in richness estimates were seen in sites characterized by clear, fast‐flowing water. This is related to the increased occupancy probabilities of many species in this habitat, as species that expressed this relationship were more likely to cross thresholds in clear‐water sites without doing so in the sites within the bog area. These results demonstrate that, even in a relatively small system, variation in habitat can lead to largely different levels of species richness at the site level. Additionally, these findings suggest that certain habitats may be more prone to issues of detection. This is consistent with the idea that site‐specific characteristics can influence detectability relative to comparable samples at other sites (Iknayan et al., 2014).

### 4.3 Community structure

Community structure also underwent changes at each threshold level, with overall representation undergoing increasing change as threshold values decreased. Procrustes analyses revealed that the largest changes were consistently seen in a limited number of species: Smallmouth Bass, Brook Trout, and White Sucker. These species, although unrelated and spanning three taxonomic families, were all characterized by low detection probabilities. Interestingly, occupancy probabilities were highly variable among these species, ranging from close to zero to just below one. This suggests that difficult‐to‐detect species may be the most influential in determining our understanding of community structure through ordination analyses, regardless of occurrence or rarity. Procrustes analyses also revealed a number of sites that exhibited large changes across the thresholds. In general, sites characterized by clear, fast‐flowing waters underwent larger changes than those in the bog habitat, with sites 30 and 31 consistently displaying large changes. Each of these sites contained at least one species characterized by low detection. This suggests that sites or habitats with many species characterized by low detection probabilities may have the potential to contribute more significantly to our overall understanding of community structure than previously thought.

These findings, combined with other published studies (e.g., Bailey, Simons, & Pollock, 2004; Kery & Schmidt, 2008; MacKenzie, 2005; Zipkin et al., 2009), suggest that standard datasets, obtained through ecological sampling, are likely missing information about species occurrences across sampling locations and underestimate the composition of species at any given location. This issue may be especially pertinent for aquatic communities, as these systems present challenges in sampling that are often not encountered in terrestrial systems. For example, visual surveys are often made impossible in aquatic systems by environmental factors specific to these ecosystems, such as water clarity and depth. Other factors, such as water flow, can make sampling in these systems logistically more difficult. Imperfect detection may thus be playing a larger role in driving our understanding of aquatic communities than previously thought. This is particularly true for studies focusing on communities that contain large numbers of difficult‐to‐detect species. Imperfect detection may also have impacts beyond the field of aquatic community ecology. In this study, variability in detection was shown to largely impact estimates of species richness. This has serious implications for conservationists, as these estimates are frequently used as a basis for both decision and policy making (Yoccoz et al., 2001). Multivariate community studies consider species composition at each site—more detailed information than simply species richness. As a result, such community ordination analyses will be impacted to a greater degree by underestimation of species composition at each of the sampling locations. Errors in these estimates of richness and composition may also affect population and game management.

Despite increasing in popularity, multispecies occupancy models have had limited use to date, particularly in a fish community context. A shortcoming in the use of such models is a lack of understanding regarding the overall effect they may have on community analyses, how sensitive the outcomes may be due to selection of different thresholds and ultimately, how our interpretations of community structure may be impacted. We have combined the use of different thresholds in multispecies occupancy models with commonly used multivariate ordination methods and demonstrated how the use of Procrustes analysis can provide insight into such choices and which species and sampling locations may be most susceptible to impacts of imperfect detection. Our approach provides a means to determine the robustness of our community sampling and the analyses conducted on these data.

## CONFLICT OF INTEREST

None declared.

## AUTHOR CONTRIBUTIONS

Mark Ridgway designed the sampling methodology, and David Benoit and Mark Ridgway collected the data. David Benoit and Donald Jackson analyzed the data. David Benoit led the writing of the manuscript. All authors contributed substantially to the drafts.

## DATA ACCESSIBILITY

Data are accessible on Dryad repository: https://doi.org/10.5061/dryad.ft71172.
