# Supplementary material for: Assessing the impacts of imperfect detection on estimates of diversity and community structure through multispecies occupancy modeling
Source: Ecol Evol. 2018 Apr 15;8(9):4676–84. doi: 10.1002/ece3.4023 (PMC5938444; doi:10.1002/ece3.4023)
Supplement: Supplementary file 1 [file ECE3-8-4676-s001.pdf]

Table S1: Catch data for the July 2009 survey of Costello Creek. Catch counts were calculated by summing the total number of individuals caught in each of the three temporal replicates.

	Species											
Site	Brook Stickleback	Brook Trout	Brown Bullhead	*Chrosomus spp.*	Common Shiner	Creek Chub	Golden Shiner	Northern Pearl Dace	Pumpkinseed	Smallmouth Bass	White Sucker	Yellow Perch
1	0	0	83	0	0	27	43	0	1	0	1	5
2	0	0	130	0	0	3	8	0	0	0	0	0
3	0	0	95	0	1	8	8	0	0	0	0	0
4	0	0	84	0	1	10	25	0	3	0	0	3
5	0	0	92	0	0	0	10	0	1	0	3	5
6	0	0	118	0	0	5	3	0	0	0	0	3
7	0	0	113	0	0	12	3	0	0	0	0	1
8	0	0	3	0	0	43	3	0	0	0	0	0
9	0	0	60	0	7	36	2	0	0	0	1	2
10	0	0	18	0	33	104	7	0	2	0	1	12
11	0	0	25	0	25	50	3	0	3	0	1	18
12	0	0	27	0	9	24	101	0	6	0	0	14
13	0	0	42	0	39	36	29	2	4	0	2	20
14	0	0	26	0	28	71	14	1	1	0	1	15
15	0	0	34	0	31	45	27	0	9	0	0	30
16	0	0	2	6	25	88	95	23	56	0	0	52
17	0	1	6	2	15	100	15	0	10	0	0	9
18	0	0	2	0	15	66	1	0	0	0	0	18
19	0	0	0	0	29	54	5	0	1	0	0	16
20	0	0	11	1	11	80	8	4	8	0	0	15
21	0	5	0	36	23	176	0	15	9	0	0	35
22	0	0	3	0	16	126	0	4	13	0	3	29
23	0	0	14	0	3	86	1	1	2	0	0	12
24	0	0	39	0	57	51	7	0	4	0	0	21
25	0	0	1	7	232	193	0	0	2	0	0	14
26	0	0	3	2	7	42	0	2	1	1	0	13
27	0	0	14	0	28	154	1	2	2	0	0	17
28	0	0	13	0	15	45	0	0	4	0	0	2
29	0	0	2	0	16	77	0	0	1	0	0	5
30	1	0	17	1	19	14	0	0	2	0	0	15
31	0	0	0	0	5	60	0	0	13	0	1	1

Table S2: Catch data for the July 2015 survey of Costello Creek. Catch counts were calculated by summing the total number of individuals caught in each of the three temporal replicates.

	Species											
Site	Brook Stickleback	Brook Trout	Brown Bullhead	*Chrosomus spp.*	Common Shiner	Creek Chub	Golden Shiner	Northern Pearl Dace	Pumpkinseed	Smallmouth Bass	White Sucker	Yellow Perch
1	0	0	33	0	1	37	35	0	12	0	0	21
2	0	0	55	0	2	33	12	0	2	0	0	17
3	1	0	43	0	4	12	10	0	23	0	0	2
4	0	0	99	0	2	15	11	0	17	0	0	7
5	0	0	119	0	0	8	4	0	4	0	0	8
6	0	0	20	0	3	18	13	0	40	0	0	5
7	0	0	22	1	1	40	16	14	12	0	0	15
8	0	0	43	0	4	29	45	0	3	0	1	3
9	0	0	26	1	14	75	31	0	5	0	0	17
10	0	0	12	0	35	105	7	0	1	0	0	16
11	0	0	81	0	3	35	2	0	0	0	1	22
12	0	0	6	0	7	68	29	0	4	0	0	10
13	0	0	7	5	6	45	9	1	26	0	0	18
14	0	0	8	1	8	115	29	1	4	0	0	51
15	0	0	2	1	9	100	46	12	14	0	0	20
16	0	0	0	6	1	139	16	7	13	0	0	37
17	0	0	3	0	29	78	11	1	0	0	2	20
18	0	0	11	0	17	92	5	0	1	0	0	20
19	0	1	1	0	18	87	5	1	7	0	0	37
20	1	0	0	50	9	156	0	0	6	0	0	8
21	0	1	0	1	33	162	0	27	0	0	0	5
22	0	0	0	33	9	305	0	1	3	0	0	13
23	8	0	2	95	3	54	0	0	1	0	0	1
24	0	0	13	0	8	100	53	1	3	0	1	26
25	0	0	0	21	50	116	0	0	2	0	0	2
26	0	0	0	0	10	105	0	0	0	0	0	36
27	0	0	23	6	51	126	12	10	2	0	3	24
28	1	0	13	64	9	127	9	0	2	0	0	6
29	0	0	9	18	6	182	0	0	7	0	0	19
30	0	0	0	0	16	71	0	0	7	2	1	14
31	0	0	2	0	0	34	0	0	41	0	0	2

Table S3: Occupancy probability values produced by the model for each species at each site.

	Species										
Site	AMNE	CUIN	SAFO	SEAT	CHSP	LUCO	NOCR	LEGI	MANA	MIDO	CACO	PEFL
1	0.9976	0.7387	0.1514	0.9999	0.0336	0.9911	0.9995	0.9999	0.1519	0.0005	0.9979	0.9999
2	0.9971	0.6877	0.0648	0.9999	0.0297	0.9809	0.9997	0.9999	0.0704	0.0002	0.9962	0.9999
3	0.9970	0.6939	0.0626	0.9999	0.0356	0.9790	0.9996	0.9999	0.0739	0.0002	0.9962	0.9998
4	0.9970	0.7311	0.0765	0.9999	0.0662	0.9778	0.9992	0.9999	0.1129	0.0004	0.9968	0.9999
5	0.9968	0.7506	0.0744	0.9999	0.1088	0.9723	0.9986	0.9999	0.1360	0.0006	0.9969	0.9999
6	0.9966	0.7535	0.0618	0.9999	0.1427	0.9634	0.9983	0.9999	0.1322	0.0006	0.9966	0.9998
7	0.9959	0.7175	0.0267	0.9998	0.1570	0.9220	0.9988	0.9998	0.0706	0.0003	0.9943	0.9998
8	0.9962	0.7678	0.0533	0.9999	0.2292	0.9488	0.9971	0.9998	0.1465	0.0008	0.9964	0.9998
9	0.9967	0.8196	0.1259	0.9999	0.3236	0.9723	0.9926	0.9999	0.3194	0.0027	0.9980	0.9999
10	0.9968	0.8477	0.1819	0.9999	0.4411	0.9770	0.9847	0.9999	0.4579	0.0061	0.9985	0.9999
11	0.9965	0.8474	0.1456	0.9999	0.5164	0.9680	0.9817	0.9999	0.4347	0.0058	0.9983	0.9999
12	0.9958	0.7987	0.0492	0.9998	0.4495	0.9223	0.9922	0.9998	0.1990	0.0014	0.9965	0.9998
13	0.9969	0.8941	0.3261	0.9999	0.7166	0.9819	0.9294	0.9999	0.7312	0.0299	0.9992	0.9999
14	0.9970	0.9101	0.4057	0.9999	0.8063	0.9838	0.8690	0.9999	0.8192	0.0586	0.9993	0.9999
15	0.9969	0.9162	0.4304	0.9999	0.8443	0.9837	0.8276	0.9999	0.8472	0.0769	0.9994	0.9999
16	0.9969	0.9234	0.4470	0.9999	0.8907	0.9821	0.7527	0.9999	0.8752	0.1067	0.9994	0.9999
17	0.9969	0.8996	0.3488	0.9999	0.7507	0.9823	0.9127	0.9999	0.7619	0.0372	0.9992	0.9999
18	0.9970	0.9083	0.3963	0.9999	0.7965	0.9837	0.8782	0.9999	0.8101	0.0542	0.9993	0.9999
19	0.9971	0.9213	0.5050	0.9999	0.8420	0.9875	0.8084	0.9999	0.8766	0.1001	0.9995	0.9999
20	0.9971	0.9254	0.5200	0.9999	0.8692	0.9871	0.7651	0.9999	0.8917	0.1217	0.9995	0.9999
21	0.9966	0.8995	0.2758	0.9999	0.8176	0.9728	0.8910	0.9999	0.7403	0.0353	0.9991	0.9999
22	0.9970	0.9327	0.5363	0.9999	0.9146	0.9855	0.6580	0.9999	0.9146	0.1724	0.9996	0.9999
23	0.9966	0.9242	0.3923	0.9999	0.9184	0.9757	0.7084	0.9999	0.8697	0.1074	0.9994	0.9999
24	0.9970	0.8946	0.3446	0.9999	0.7012	0.9836	0.9318	0.9999	0.7386	0.0307	0.9992	0.9999
25	0.9967	0.9419	0.5388	0.9999	0.9585	0.9809	0.4632	0.9999	0.9380	0.2677	0.9996	0.9999
26	0.9967	0.9468	0.5562	0.9999	0.9714	0.9794	0.3583	0.9999	0.9500	0.3412	0.9996	0.9999
27	0.9959	0.9312	0.2869	0.9999	0.9707	0.9470	0.4999	0.9999	0.8774	0.1400	0.9992	0.9999
28	0.9952	0.9199	0.1568	0.9999	0.9732	0.8985	0.5703	0.9999	0.7945	0.0741	0.9988	0.9999
29	0.9960	0.9505	0.4386	0.9999	0.9885	0.9571	0.2112	0.9999	0.9503	0.3866	0.9995	0.9999
30	0.9962	0.9595	0.5697	0.9999	0.9925	0.9678	0.1167	0.9999	0.9731	0.5934	0.9997	0.9999
31	0.9954	0.9441	0.2957	0.9999	0.9896	0.9262	0.2464	0.9999	0.9217	0.2664	0.9993	0.9999

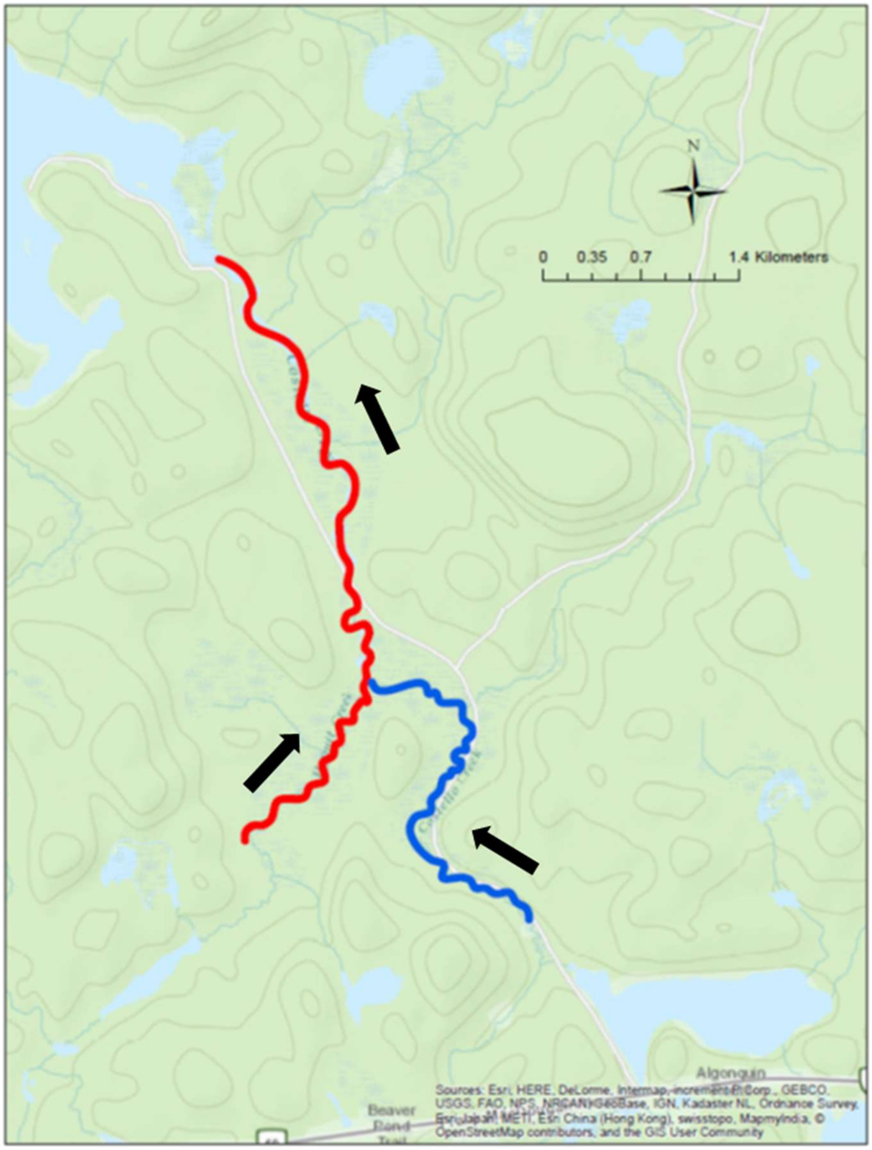

Figure S1: Costello Creek with Habitat A (draining a bog area) depicted in red and Habitat B (clear, fast flowing) depicted in blue, with arrows representing the direction of flow.

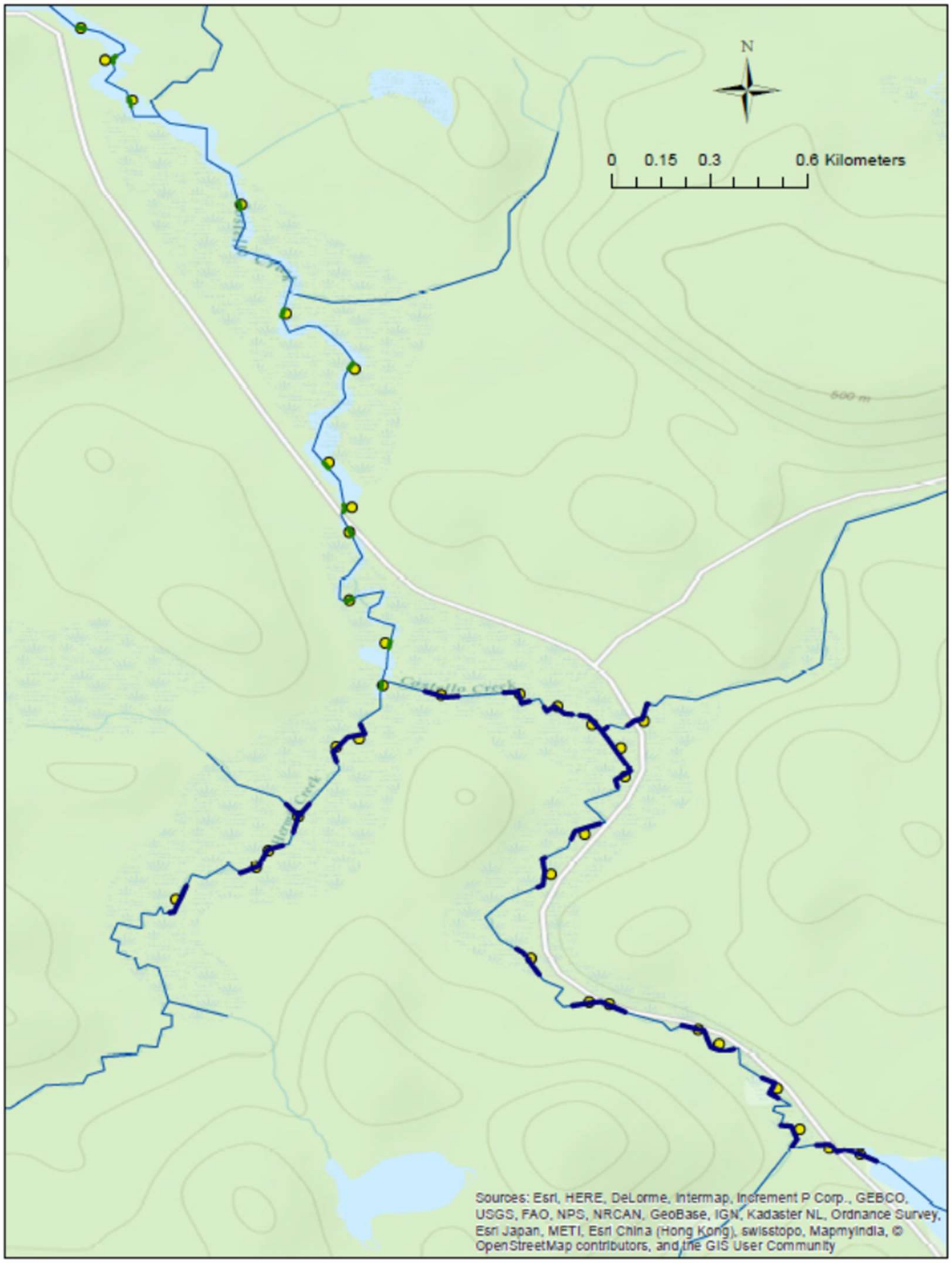

Figure S2: Home range of the largest fish caught in the system surrounding each site. Home ranges in the upper stream are depicted in blue and ranges in the lower stream are depicted in green.

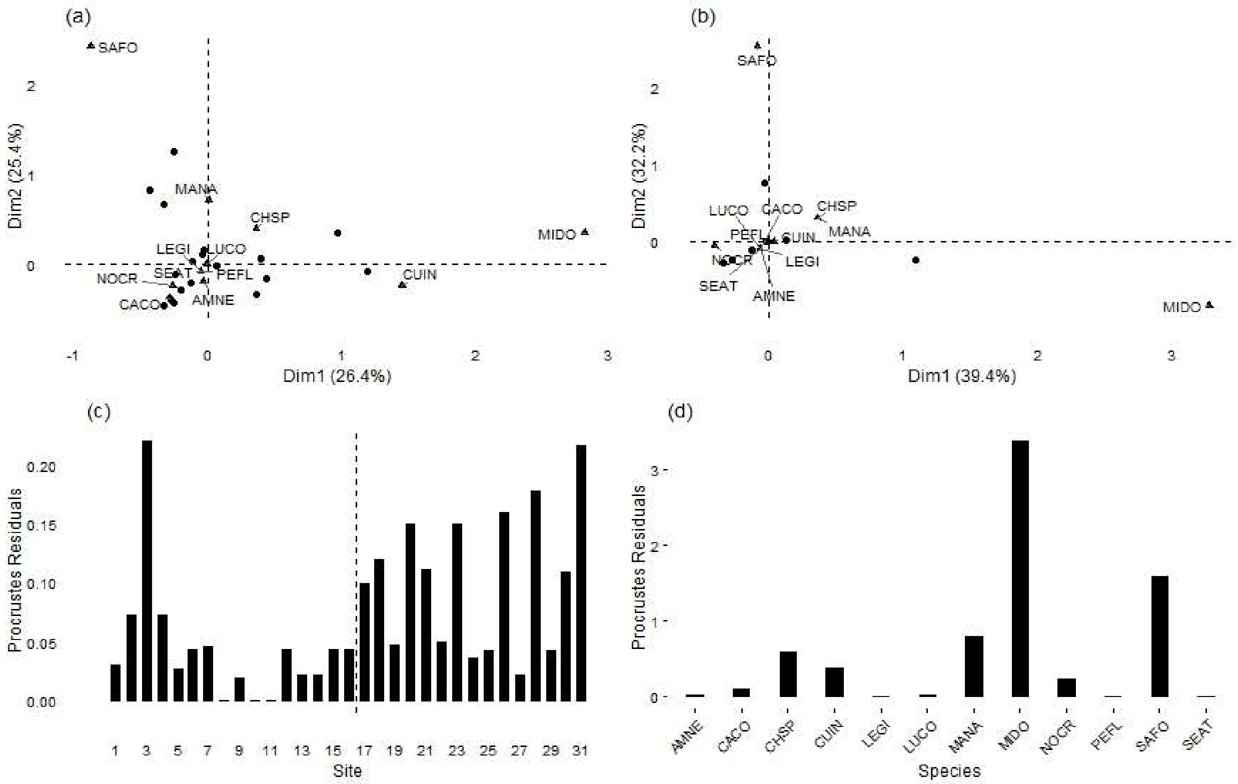

Figure S3: Results of the correspondence analysis using: a) the standard dataset of fish species presence-absence in Costello Creek and; b) a dataset informed at the 75% occupancy threshold. Changes between the two ordinations are displayed as Procrustes residuals for c) sites with a dashed line distinguishing the slow-moving, bog habitat (on the left) from the clearer, faster-flowing waters (on the right) and d) each species.

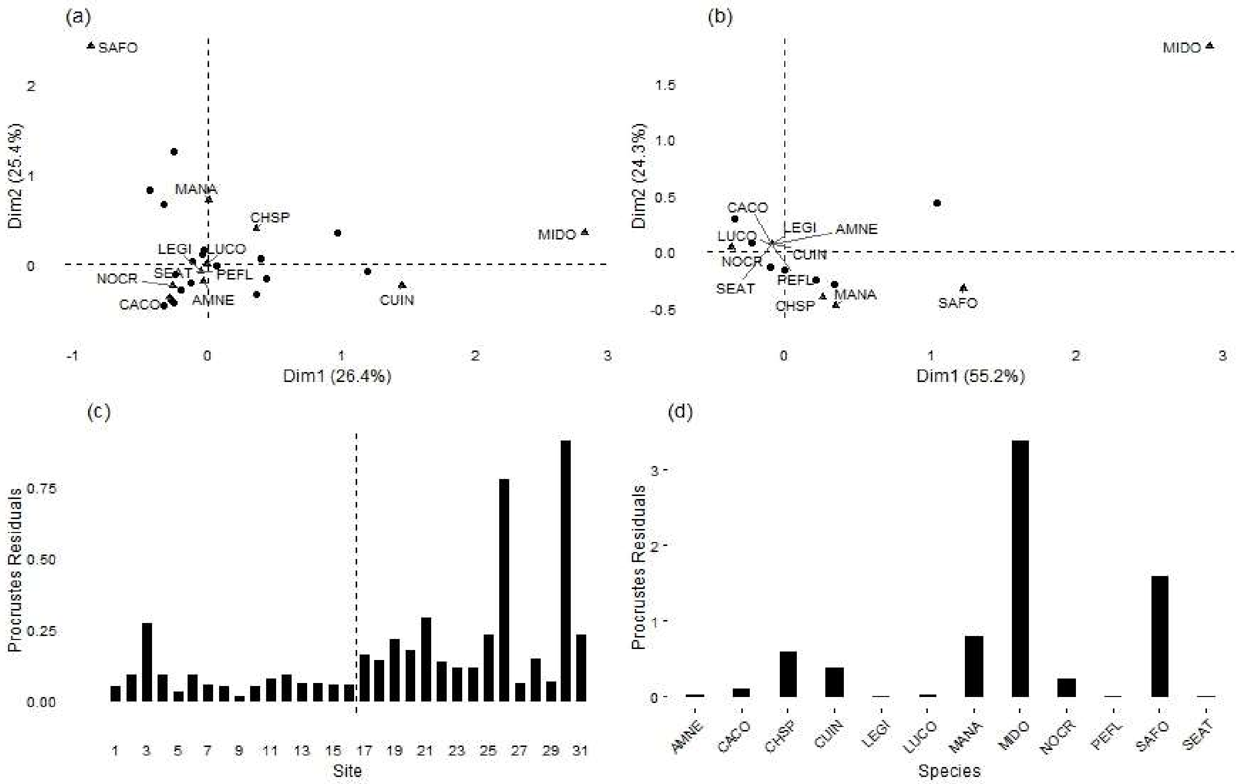

Figure S4: Results of the correspondence analysis using: a) the standard dataset of fish species presence-absence in Costello Creek and; b) a dataset informed at the 50% occupancy threshold. Changes between the two ordinations are displayed as Procrustes residuals for c) sites with a dashed line distinguishing the slow-moving, bog habitat (on the left) from the clearer, faster-flowing waters (on the right) and d) each species.
